# Brake wear particle emissions: a review

**DOI:** 10.1007/s11356-014-3696-8

**Published:** 2014-10-17

**Authors:** Theodoros Grigoratos, Giorgio Martini

**Affiliations:** European Commission, Joint Research Centre, Sustainable Transport Unit (STU), Institute for Energy and Transport (IET), Via E Fermi 2749, 21027 Ispra, Italy

**Keywords:** Non-exhaust emissions, Brake wear particles, Size distribution, Chemical composition, Emission factors, Health relevance

## Abstract

Traffic-related sources have been recognized as a significant contributor of particulate matter particularly within major cities. Exhaust and non-exhaust traffic-related sources are estimated to contribute almost equally to traffic-related PM_10_ emissions. Non-exhaust particles can be generated either from non-exhaust sources such as brake, tyre, clutch and road surface wear or already exist in the form of deposited material at the roadside and become resuspended due to traffic-induced turbulence. Among non-exhaust sources, brake wear can be a significant particulate matter (PM) contributor, particularly within areas with high traffic density and braking frequency. Studies mention that in urban environments, brake wear can contribute up to 55 % by mass to total non-exhaust traffic-related PM_10_ emissions and up to 21 % by mass to total traffic-related PM_10_ emissions, while in freeways, this contribution is lower due to lower braking frequency. As exhaust emissions control become stricter, relative contributions of non-exhaust sources—and therefore brake wear—to traffic-related emissions will become more significant and will raise discussions on possible regulatory needs. The aim of the present literature review study is to present the state-of-the-art of the different aspects regarding PM resulting from brake wear and provide all the necessary information in terms of importance, physicochemical characteristics, emission factors and possible health effects.

## Introduction

Particles emitted as a result of road transport activity can be distinguished according to their source into exhaust traffic-related particles, which are emitted as a result of incomplete fuel combustion and lubricant volatilization during the combustion procedure, and non-exhaust traffic-related particles, which are either generated from non-exhaust traffic-related sources or already exist in the environment as deposited material and become resuspended due to traffic-induced turbulence. Exhaust particles have been very well studied and characterized, while technological improvements have resulted in a significant reduction of their emissions (Amato et al. 2014; Denier Van der Gon et al. 2013; Pant and Harrison 2013). On the other hand, non-exhaust processes have not yet been adequately studied, and several questions regarding physicochemical characteristics, emission factors and possible adverse health effects of wear particles still remain unanswered (Denier Van der Gon et al. 2013). The most important abrasion processes which result in direct particulate matter (PM) emission are tyre, brake, clutch and road surface wear, with other potential sources being engine wear, abrasion of wheel bearings and corrosion of other vehicle components, street furniture and crash barriers (Barlow et al. 2007; Boulter 2006; Pant and Harrison 2013; Thorpe and Harrison 2008). Besides direct traffic-related sources, non-exhaust PM exists due to resuspension of material already deposited on the road surface as a result of tyre shear, vehicle-generated turbulence, and the action of the wind. Road dust resuspension can be a significant contributor of PM especially in dryer climates (Abu-Allaban et al. 2003; Amato et al. 2009; Amato et al. 2010).

In the case of road transport, there is a general conviction that most primary fine particles are emitted from the exhaust, whereas many of the coarse particles are considered to originate from non-exhaust sources. This is not precise since there is much evidence that non-exhaust particles contribute to both the fine and coarse modes of PM_10_ (Amato et al. 2014; Boulter 2006; Dahl et al. 2006; Denier Van der Gon et al. 2013; Gustafsson et al. 2008; Harrison et al. 2012; Kukutschová et al. 2011; Kumar et al. 2013; Mathissen et al. 2011). There are numerous studies reporting that exhaust and non-exhaust traffic-related sources contribute almost equally to total traffic-related PM_10_ emissions, while due to continuous reduction of exhaust emissions, it is expected that the relative contribution of non-exhaust sources will increase in the forthcoming years (Amato et al. 2011; Amato et al. 2014; Bukowiecki et al. 2009a; Denby et al. 2013; Denier Van der Gon et al. 2013; Ketzel et al. 2007; Pant and Harrison 2013; Querol et al. 2004). One of the most important non-exhaust traffic-related source is considered to be brake wear, with studies at urban environments reporting contribution to non-exhaust traffic-related PM_10_ emissions up to 55 % by mass (Harrison et al. 2012) and to total traffic-related PM_10_ emissions up to 21 % by mass (Bukowiecki et al. 2009a; Gasser et al. 2009; Lawrence et al. 2013). On the other hand, it is more difficult to extract precise estimations on the contribution of brake wear to ambient PM_10_ concentrations since this contribution depends on various other parameters besides the sampling location. However, there are studies which have revealed brake wear contributions to ambient PM_10_ concentrations varying from negligible to up to 4 μg m^−3^ (Amato et al. 2009; Bukowiecki et al. 2009a; Denier Van der Gon et al. 2007; Furusjö et al. 2007; Harrison et al. 2012; Sjödin et al. 2010; Wåhlin et al. 2006).

Several difficulties arise when studying particles from non-exhaust traffic-related sources and particularly from brake wear. Probably the most important restriction has to do with the lack of standardized sampling procedure and measurement techniques. This often leads in different experimental approaches by researchers and therefore in non-comparable results and conclusions. Several studies have shown that brake wear debris differs depending on the bulk friction material (Kukutschová et al. 2011; Österle et al. 2001). Additionally, brake wear particles’ chemical composition and emission rates largely depend on the driving behaviour and more particularly on the frequency and severity of braking (Kwak et al. 2013). Vehicle’s speed, condition and maintenance history can also be important parameters. Finally, the conditions under which the braking event occurs (ambient temperature and chemicals available in the environment) can have a big impact on the characteristics of the generated particles (Barlow et al. 2007; Boulter 2006; Boulter et al. 2006; Kukutschová et al. 2011; Mosleh et al. 2004; Olofsson and Olander 2013).

Despite the difficulties in measuring and characterizing brake wear particles, an increasing number of researchers and experts have already raised a discussion on the need for regulating emissions from non-exhaust sources including brake wear (Amato et al. 2014; Denier Van der Gon et al. 2013). The aim of the present literature review is to present the state-of-the-art of the different aspects regarding PM resulting from brake wear and provide all the necessary information in terms of importance, physicochemical characteristics, emission factors and possible adverse health effects. Also, this study aims in identifying the major gaps regarding the above-mentioned issues since it is necessary to take them into account in order to assess the possible need for regulating brake wear emissions.

## General information and importance of brake wear particles

**Fig. 1:**
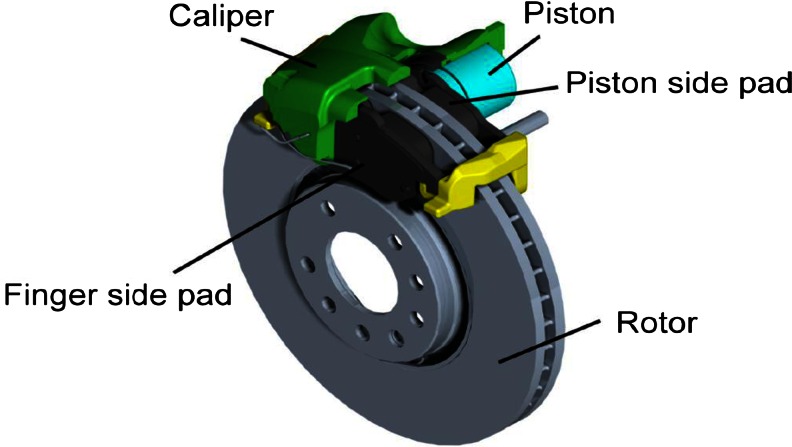
Graphic representation of a disc brake system. Source: [Wahlström 2009]

Two brake system configurations have been widely used in modern passenger vehicles: disc brakes, in which flat brake pads are forced against a rotating metal disc (Fig. 1), and drum brakes, in which curved brake shoes are forced against the inner surface of a rotating cylinder. Modern passenger vehicles are usually equipped with disc front and rear brakes, while in the past, drum brakes were usually employed as rear brakes. It is estimated that front brakes have to provide approximately 70 % of total braking power and therefore have to be replaced more frequently than rear ones. The majority of car braking systems consist of frictional pairs made of a disc, a pad and a calliper. Figure 1 depicts a disc brake assembly with a single-piston floating calliper and a ventilated rotor (Wahlström 2009). Rotors used in passenger vehicles are usually made of grey cast iron, but in some cases, they can be made of composites such as reinforced carbon–carbon, ceramic matrix composites and aluminum.

Brake linings generally comprise five main components: binders, fibres, fillers, frictional additives or lubricants and abrasives (Boulter 2006; Chan and Stachowiak 2004; Kukutschová et al. 2011; Thorpe and Harrison 2008; Wahlström 2009; Wahlström 2011). Binders hold the components of the brake pad together and ensure the structural integrity of the lining under mechanical and thermal stress. They account for 20–40 % of the lining material and are made of modified phenol-formaldehyde resins. Reinforcing fibres provide mechanical strength and structure to the lining. They usually account for 6–35 % (by mass) of the lining material and can be classified as metallic, mineral, ceramic or organic. They mainly consist of copper, steel, brass, potassium titanate, glass, organic material and Kevlar. Fillers are used in order to improve thermal and noise pad properties and also reduce the manufacturing cost. They usually consist of inorganic compounds (barium and antimony sulphate, magnesium and chromium oxides), silicates, ground slag, stone and metal powders and account between 15 and 70 % (by mass) of the lining material. Lubricants influence the wear characteristics of the lining. They can be inorganic, metallic or organic. Graphite is usually employed, but other common materials include ground rubber, metallic particles, carbon black, cashew nut dust and antimony trisulphide. They usually make up 5–29 % by mass of the brake lining. Abrasives are used in order to increase friction, maintain cleanliness between contact surfaces and limit the buildup of transfer films. They typically account for up to 10 % by mass of the lining. Aluminum oxide, iron oxides, quartz and zircon are the most common abrasive constituents. The proportions of the above-mentioned components vary according to the type of lining and the manufacturer (Boulter et al. 2006; Eriksson et al. 2001). Three different lining types are usually found in passenger vehicles: non-asbestos organic (NAO), semimetallic and low metallic. NAO-type pads are relatively soft and exhibit low brake noise compared to other types of pads, but they lose braking capacity at high temperature and create more dust than the other types. For many years, brake linings were composed of asbestos fibres, while today, they are asbestos free due to serious health concerns (Lemen 2004; Liew and Nirmal 2013; Roubicek et al. 2008; Thorpe and Harrison 2008). Low-metallic pads comprise organic compounds mixed with small amounts of metals (10–30 % by mass). They exhibit high friction and good braking capacity at high temperatures. Semimetallic brake pads have higher metallic content (up to 65 % by mass), which makes them more durable and with excellent heat transfer. On the other hand, they tend to wear down rotors faster and exhibit intrusive noise characteristics. For high performance requirements, or extreme braking conditions (sports cars, ambulances, police cars), metallic linings which contain steel and copper fibres are employed.

**Fig. 2:**
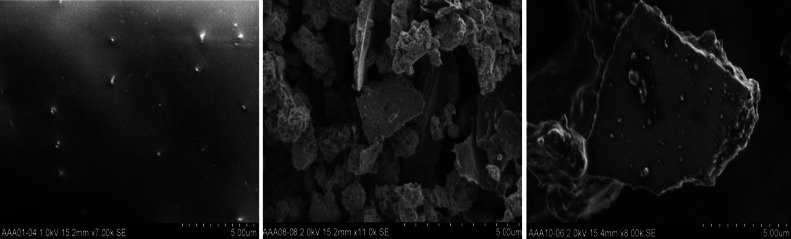
SEM images of brake wear particles (left <56 nm, middle PM_2.5_, right PM_10_). Source: [Kukutschová et al. 2011]

**Fig. 3:**
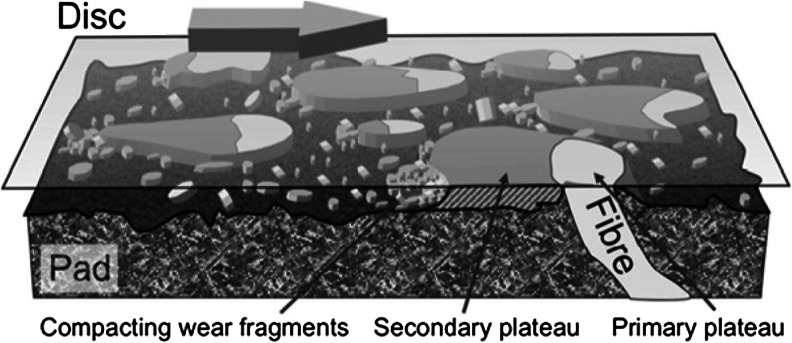
Illustration of the contact situation between the pad and disc. A transparent disc is moving from left to right. Some of the wear particles pile up against the contact plateaus and create secondary plateaus. A flow of wear particles in the gap between the pad and disc wear the lowlands of the pad through three-body abrasion. Source: [Wahlström 2011]

The frictional contact between the disc and the pad generates particles of various sizes. During a braking event, the calliper acts mechanically on the pad, which slides against the disc and transforms vehicle kinetic energy into thermal energy. Apart from the mechanical abrasion, vehicle brakes become subject to large frictional heat generation with subsequent wear of linings and rotors. This generates mostly micron-sized particles. Figure 2 shows particles of different sizes generated as a result of brake wear tests performed in the laboratory (Kukutschová et al. 2011). Finally, some disc-brake systems require the pads to be in low-pressure contact with the rotor in order to ensure robust brake performance. This leads in higher particle release in the environment (Söderberg et al. 2008). A detailed explanatory model of the complex contact situation between an organic brake pad and a cast iron disc has been developed and published by some researchers (Eriksson et al. 2002; Österle et al. 2001; Ostermeyer 2001). In this model, the macroscopic friction and wear behaviour of a disc brake can be explained by the microscopic contact situation (growth and destruction of contact plateaus) in the boundary layer between the pad and disc. Wahlström (2011) provided a simplified visual explanation of the model which is given in Fig. 3. It has been shown that the plateau surface is covered by a nanocrystalline third body formed by the wear particles and that this third body is mainly made of iron oxides (Österle and Urban 2006). The third body differs in structure composition and properties from the two first bodies, pad and disc in our case (Oesterle and Dmitriev 2014). Much detailed work has been published on this field by several researchers (Bodel and Ostermeyer 2014; Ostermeyer 2007; Ostermeyer and Muller 2008).

Not all wear debris generated during braking become airborne. Approximately 50 % of wear particles lie into diameters smaller than 20 μm (Barlow et al. 2007; Garg et al. 2000; Kukutschová et al. 2011; Sanders et al. 2003), while almost 40 % of brake wear debris is emitted as PM_10_ (Garg et al. 2000; Harrison et al. 2012; Kumar et al. 2013; Kukutschová et al. 2011; Mosleh et al. 2004; Sanders et al. 2003; Wik and Dave 2009). The rest may deposit on the road surface or be attracted to the vehicle with its fate remaining unknown. It is easily understood that the highest concentrations of brake wear particles are observed near busy junctions, traffic lights, pedestrian crossings and corners, even if wear particles may also be released from the brake mechanism some time after the primary emission event (Kennedy et al. 2002). Several studies at urban environments have reported contribution of brake wear particles to non-exhaust traffic-related PM_10_ emissions ranging between 16 and 55 % by mass (Bukowiecki et al. 2009a; Gasser et al. 2009; Harrison et al. 2012; Lawrence et al. 2013; Riediker et al. 2008), while significantly lower contributions (∼3 % by mass) have been reported in highways where braking events are less frequent (Abu-Allaban et al. 2003; Bukowiecki et al. 2009a). Additionally, studies have revealed contributions of brake wear to total traffic-related PM_10_ emissions of 11–21 % by mass (Bukowiecki et al. 2009a; Gasser et al. 2009; Lawrence et al. 2013). On the other hand, brake wear contributions to ambient PM_10_ concentrations vary from negligible to up to 4 μg m^−3^ (Amato et al. 2009; Bukowiecki et al. 2009a; Denier Van der Gon et al. 2007; Harrison et al. 2012; Wåhlin et al. 2006). Taking into consideration the general decreasing tendency of engine exhaust emissions due to the use of catalytic converters, diesel particulate filters (DPF) and improved fuels and engines, it is expected that the relative contribution of brake wear particles to the total PM levels will increase in the forthcoming years making the need for characterization of these particles urgent (Amato et al. 2014; Denier Van der Gon et al. 2013; Pant and Harrison 2013; Wik and Dave 2009).

## Brake wear particle mass distribution

Although brake wear particles are emitted as a result of a predominantly mechanical process and are expected to lie mainly into the coarse size fraction, there are many studies which have shown high particle concentrations also in the fine and ultrafine fractions (Garg et al. 2000; Kukutschová et al. 2011; Kwak et al. 2013; Iijima et al. 2007; Riediker et al. 2008; Sanders et al. 2003; von Uexküll et al. 2005; Wahlström et al. 2010b). SEM images of brake wear particles demonstrated that ultrafine and some of the fine particles seem to be smoother with fewer sharp edges compared to bigger particles, thus pointing to thermal and/or chemical generation processes (Wahlström et al. 2010a). This can be a result of high temperatures at the brake/rotor interface which lead in the decomposition of brake lining materials. Garg et al. (2000) tested different brake pads and found that 86 and 63 % of the brake wear airborne particle mass was distributed in the PM_10_ and PM_2.5_, respectively. Surprisingly, a considerable 33 % (by mass) of wear particles was found into diameters smaller than 0.1 μm. Similarly, Sanders et al. (2003) tested different brake pads and found that PM_10_ accounted for 63–85 % of the total brake wear mass, depending on the type of pad. Iijima et al. (2008) reported that 56–70 % of total brake wear mass generated from three different NAO pads was emitted as PM_2.5_, while 95–98 % was emitted as PM_10_. Similar distributions (98 and 39 % for PM_10_ and PM_2.5_, respectively) are extracted by the EMEP/CORINAIR Emissions Inventory Guidebook (Thorpe and Harrison 2008). On the other hand, receptor modelling studies showed that brake wear contribution was significantly higher in PM_10_ than in PM_2.5_ fraction (Abu-Allaban et al. 2003).

**Fig. 4:**
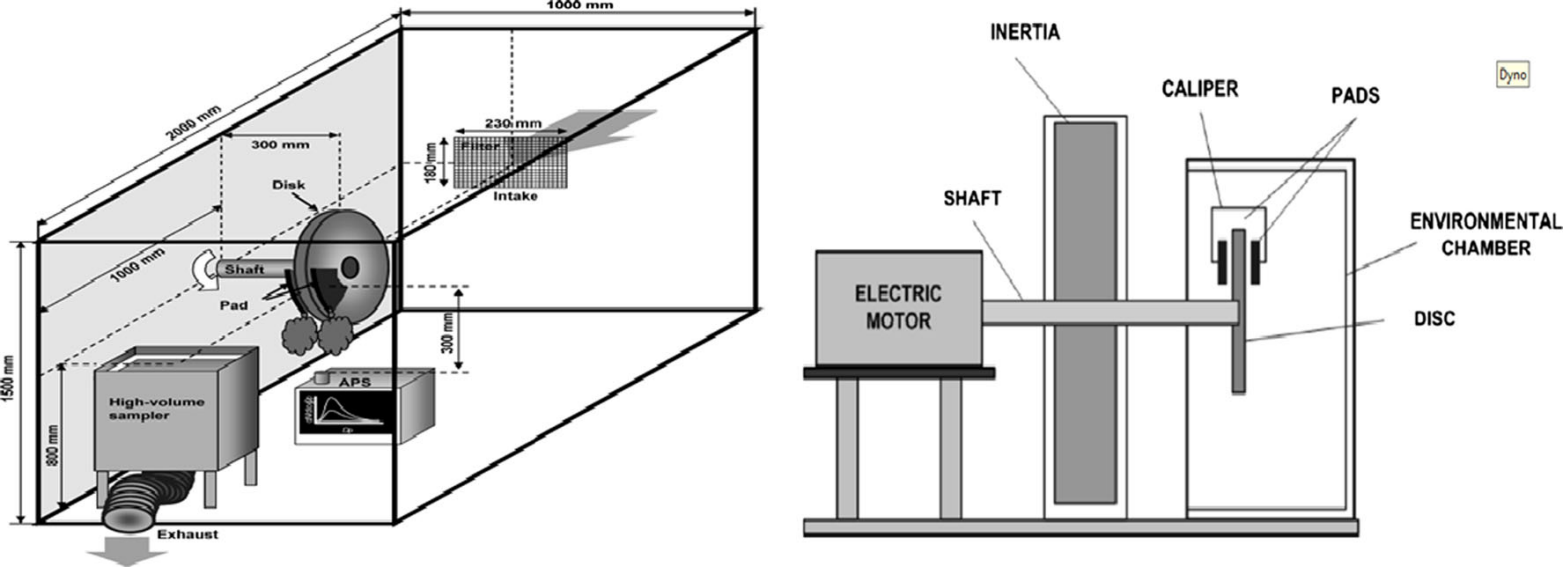
Schematic representation of the brake dynamometer assembly. Sources: [*left* Iijima et al. 2008; *right* Kukutschová et al. 2011]

Studies dealing with brake wear particle characterization can be distinguished in those which the collection and characterization of particles take place in the laboratory by means of brake dynamometer tests (Garg et al. 2000; Gasser et al. 2009; Kukutschová et al. 2011; Iijima et al. 2007; Iijima et al. 2008; Mosleh et al. 2004; Sanders et al. 2003; Sondhi et al. 2010; Wahlström et al. 2010b), those which the collection and characterization of particles are performed in ambient air and then brake wear emissions are identified by means of specific brake wear tracers (Bukowiecki et al. 2009a; Bukowiecki et al. 2009b; Dongarra et al. 2009; Gietl et al. 2010; Harrison et al. 2012; Hjortenkrans et al. 2007; Fabretti et al. 2009; Sondhi et al. 2010; Wåhlin et al. 2006) and those which brake wear particles are directly sampled on-road under “real-world” driving conditions by means of mobile units (Mathissen et al. 2011). In Fig. 4, a schematic representation of the assembly used in brake dynamometer studies is provided (Kukutschová et al. 2011; Iijima et al. 2008). On the right, an open sampling system is depicted, while on the left, the sampling system is closed. Table 1 provides an overview of the most important literature studies dealing with the characterization of brake wear particles in terms of their mass size distribution.

**Table 1:** Overview of literature studies investigating the mass distribution of airborne brake wear particles

Reference	Type of study	Brake pads tested	Method	Mass size distribution
Cha et al. 1983	Brake dynamometer	Asbestos		Unimodal (2.1–3.3 μm)
Garg et al. 2000	Brake dynamometer	Semimetallic and NAO	MOUDI (>0.1 μm)	Unimodal (0.1–1.0 μm)
Sanders et al. 2003	Brake dynamometer	Low metallic, semimetallic and NAO	MOUDI–ELPI	Unimodal (4–5 μm)
von Uexküll et al. 2005	Brake dynamometer	Disc and drum (trucks)	Optical particle counter (>0.3 μm)	Unimodal (2–3 μm)
Iijima et al. 2007	Brake dynamometer	NAO	APS (>0.5 μm)	Unimodal (3–6 μm)
Iijima et al. 2008	Brake dynamometer	NAO	APS (>0.5 μm)	Unimodal (2.0 μm)
Kukutschová et al. 2011	Brake dynamometer	Low metallic	APS–SMPS–BLPI	Unimodal (2–4 μm)
Harrison et al. 2012	On-road measurement	Roadside PM	MOUDI	Unimodal (2–3 μm)
Kwak et al. 2013	On-road measurement	Roadside PM	APS (>0.5 μm)	Unimodal (1–10 μm)

Most researchers have found unimodal brake wear PM_10_ mass distributions with maxima ranging between 1.0 and 6.0 μm. For instance, Sanders et al. (2003) tested low-metallic, semimetallic and NAO pads under typical urban driving conditions and found unimodal PM_10_ distribution with a mass-weighted mean diameter of 5–6 μm, while Iijima et al. (2007) tested NAO pads and found a unimodal distribution with a slightly lower mass-weighted mean diameter (3–6 μm). Both studies were carried out in an open sampling system (like the one shown in Fig. 4 on the right); therefore, the particle size distribution could not deviate from the original due to particle aggregation or deposition as a result of the interaction between the particles and the chamber wall. This effect has been described in case of close systems (Iijima et al. 2007). Iijima et al. (2007) reported that increasing disc temperature results in a slight shift of the mass distribution curve towards higher sizes. A similar observation was made by Mosleh et al. (2004) in the case of brake wear particle number distribution. Other studies also revealed unimodal mass distributions with however somewhat lower mass-weighted mean diameter. For instance, Kukutschová et al. (2011) tested low-metallic pads and found unimodal distribution with maxima at 2–4 μm, while von Uexküll et al. (2005) conducted tests on front and rear truck brakes and found unimodal mass distributions with maxima at 2–3 μm. An on-road experimental campaign conducted by Harrison et al. (2012) reached similar conclusions to brake dynamometer studies. Harrison et al. (2012) collected size-fractionated samples of airborne PM and used the size distribution of specific tracer elements in order to estimate the contribution of brake wear to particle mass. They found that brake wear particles appeared a unimodal PM_10_ mass distribution with a peak at 2–3 μm.

## Brake wear particle number distribution

Number size distribution of brake wear particles is of high importance since a significant amount of the generated particles is distributed among smaller sizes. Most researchers report at least one peak of the number size distribution at the ultrafine fraction. Garg et al. (2000) conducted brake dynamometer tests and found the highest number of emitted particles to lie into diameters smaller than 30 nm. This is in agreement with Mathissen et al. (2011) who conducted a road simulation study and found that wear particles generated at 100 km h^−1^ full stop braking events exhibited a bimodal particle number (PN) distribution with a nucleation mode at 10 nm and a second mode between 30 and 50 nm. Thirty kilometers per hour full stop braking events led to unimodal size distributions with slightly shifted maxima towards larger diameters (70–90 nm). Kukutschová et al. (2011) found that despite the fact that the generation of small wear particles (<500 nm) at low rotor temperature conditions was negligible, the concentration of nanoparticles smaller than 100 nm significantly increased with the increase of the cast iron disc temperature (up to 340 °C). They proposed that submicron particles are rather formed by the evaporation/condensation process with subsequent aggregation of primary nanoparticles than by an abrasive type of wear. Wahlström et al. (2010b) also noted a peak in the particle number distribution at approximately 100 nm, and they found a shift of the PN distribution towards lower sizes when front brakes were tested (compared to rear brakes). Riediker et al. (2008) tested pad materials of six different passenger cars under controlled environmental conditions and found a bimodal PN distribution with peaks at 80 nm (depending on the tested car and braking behaviour) and at 200–400 nm. They found that full stops result in higher nanoparticle production compared to normal deceleration. Table 2 provides an overview of the most important literature studies dealing with the characterization of brake wear particles in terms of their number distribution.

**Table 2:** Overview of literature studies investigating the number distribution of airborne brake wear particles

Reference	Type of study	Brake pads tested	Method	Particle number distribution
Sanders et al. 2003	Brake dynamometer	Low metallic, semimetallic and NA004F	ELPI	Unimodal (1.0 μm)
Mosleh et al. 2004	Brake dynamometer	Semimetallic (truck)	Laser scattering analyser	Bimodal (350 nm and 2.0 μm)
von Uexküll et al. 2005	Brake dynamometer	Disc and drum (trucks)	Optical particle counter (>0.3 μm)	Unimodal (0.5–1.0 μm)
Iijima et al. 2007	Brake dynamometer	NAO	APS (>0.5 μm)	Unimodal (1.0–2.0 μm)
Riediker et al. 2008	Brake dynamometer	Vehicles under different driving conditions	TEM	Bimodal (80 and 400 nm)
Iijima et al. 2008	Brake dynamometer	NAO	APS (>0.5 μm)	Unimodal (0.8–1.0 μm)
Wahlström et al. 2010a	Brake dynamometer	Low metallic and NAO	GRIMM (>0.25 μm)	Bimodal (280 and 350 nm)
Wahlström et al. 2010b	Brake dynamometer	Low metallic and NAO	GRIMM–SMPS	Multimodal (100–550 nm)
Mathissen et al. 2011	On-road direct measurement	Vehicle under different driving conditions	EEPS (<0.56 μm)	Bimodal (10 and 40 nm)
Kukutschová et al. 2011	Brake dynamometer	Low-metallic brake pads	APS–SMPS	Bimodal (100 and 300 nm)

Not all studies found ultrafine brake wear particles. There are several studies which show particle number distribution with the first peak at approximately 300 nm. Wahlström et al. (2010a) tested low-metallic and NAO pads and found a bimodal number size distribution with peaks at approximately 280 and 350 nm. However, this could be due to instrument’s limitation on the size of sampling particles (>0.30 μm). Mosleh et al. (2004) tested a commercial truck semimetallic pad and found a bimodal PN distribution with a peak at approximately 350 nm, regardless the sliding speed and the nominal contact pressure, and another at higher particle sizes (∼2.0 μm). They found that the second peak is related to the applied nominal contact pressure and sliding speed, and they concluded that fine wear particles originate from the cast iron disc, whereas coarse particles mainly come from the brake pad material. Wahlström et al. (2010b) used a pin-on-disc tribometer in order to test airborne wear debris generated from the interaction of NAO and low-metallic pads with grey cast iron rotors. They reported that low-metallic pads result in higher wear rates of the rotor material than NAO pads and thus in higher concentrations of airborne wear particles. In all cases, similar PN distributions with maxima at around 280, 350 and 550 nm were observed.

Finally, there are some studies which report PN distributions with the first peak at approximately 1.0 μm. For instance, Sanders et al. (2003) conducted dynamometer and on-road vehicle tests in three different classes of lining materials and found a maximum peak at approximately 1.0 μm. Iijima et al. (2007) used an APS spectrometer (measured particles >0.50 μm) in order to investigate the PN distribution from NAO brake pads and found the peak at 1.0 μm. They also observed a tendency of shifting of the distribution to coarser sizes with increasing disc temperature. Overall, brake wear particle number distributions usually appear to be bimodal with both peaks lying within the fine particle mode.

## Chemical characterization of brake wear particles

Chemical composition of brake wear particles should be taken into account when trying to fully characterize them and assess their possible adverse effects on human health. Several epidemiology studies have correlated adverse health responses with the presence of specific chemical species like carbonaceous material (Kelly and Fussell 2012; Lipfert et al. 2006; Ostro et al. 2006; WHO 2013) and trace elements (heavy metals) (Kelly and Fussell 2012; Ostro et al. 2007; Pope et al. 2007) in the ambient PM.

Modern brakes are composites of many different and sometimes unknown ingredients, and even if the chemical composition of brake wear debris significantly differs from the chemical composition of the original lining material (Kukutschová et al. 2011; Österle et al. 2001), someone has to look into both linings and wear debris composition in order to gain a comprehensive view of how the braking process affects the chemical composition of brake wear particles. Despite the large variation in the chemical composition of commercial lining materials, most researchers have reported Fe, Cu, Zn and Pb to be the most abundant metals in the brake lining (Chan and Stachowiak 2004; Figi et al. 2010; Gadd and Kennedy 2000; Kukutschová et al. 2011; Iijima et al. 2007; Schauer et al. 2006; von Uexküll et al. 2005; Westerlund 2001). Fe content can reach up to 60 wt.% and varies according to the type of lining (Chan and Stachowiak 2004; Gadd and Kennedy 2000; Kukutschová et al. 2011; Schauer et al. 2006). Cu and Zn follow at relatively high concentrations (Iijima et al. 2007; von Uexküll et al. 2005; Westerlund 2001), with K and Ti also being present at considerable concentrations. Significant Pb content (up to 12 wt.%) has been reported in older studies (Thorpe and Harrison 2008), with however latest studies showing a remarkable decrease (0.2 wt.%, Kukutschová et al. 2011) due to the replacement of Pb in modern linings (Bukowiecki et al. 2009a). Other metals such as Ba, Mg, Mn, Ni, Sn, Cd, Cr, Ti, K and Sb have also been found in concentrations lower than 0.1 wt.% (Boulter 2006; Kukutschová et al. 2011; Thorpe and Harrison 2008). Recently, brake wear emissions have been cited as a potentially important source of Sb. Brake linings contain 1–5 % Sb in the form of stibnite (Sb_2_S_3_), which is employed as a lubricant in order to reduce vibrations and improve friction stability. Roubicek et al. (2008) tested linings from different geographic regions and found European and Japanese samples to contain Sb S. Stibnite can be oxidized during the braking process to Sb_2_O_3_, which has been categorized as a potentially carcinogenic substance (Varrica et al. 2013; Von Uexküll et al. 2005). Very limited information regarding the presence of organic compounds is available in the literature (Gadd and Kennedy 2000; Rogge et al. 1993). Gadd and Kennedy (2000) examined six different commercial brake pads and found phenolic compounds to be the most abundant species present in the brake pad extracts. They also found carbonyl compounds, organic acids, methyl-esters and aromatic carboxylic acids in trace concentrations.

Chemical properties of the parent lining material are often modified due to high temperatures and pressures reached during the braking process. However, brake wear is characterized by a specific pattern of some heavy metals (Fe, Cu, Zn, Sn, Sb) in the airborne PM concentrations (Boulter 2006; Bukowiecki et al. 2009a), which are similar to those of the lining material. These elements have been extensively used as specific tracers for brake wear. Wahlström et al. (2010a) conducted a brake dynamometer study and found that fine wear particles mainly comprised Fe, Cu, Ti, Al as well as oxygen and carbonaceous species. On the other hand, coarse particles appeared as flakes and mainly comprised Fe in the form of its oxides. They concluded that these particles were mechanically generated suggesting disc wear. Some of the coarse particles also contained Ti, Cu and Al and were attributed to the brake pads. Kukutschová et al. (2011) tested low-metallic brake pads and reported that the finest brake wear PM fraction was dominated by metallic and carbonaceous species. Fe dominated metallic content in both fine and coarse fractions, while TEM analysis revealed the presence of maghemite (γ-Fe_2_O_3_), magnetite (FeO-Fe_2_O_3_) and amorphous carbon in the nanoparticle fraction and maghemite, magnetite, and hematite (α-Fe_2_O_3_) in the fine fraction. Cu, Sn, S and Zn were also present in various oxide forms. Similar results were obtained by Peikertová et al. (2013) who additionally detected MoS. Napier et al. (2008) conducted a meta-analysis of data collected in the UK and recognized brake wear as the largest single source of atmospheric Cu. Regarding carbonaceous species, carbon black was attributed to the oxidative wear and subsequent deposition from related volatiles (Yu et al. 2009), while graphitic particles were emitted as a result of abrasive wear. Similarly, Gasser et al. (2009) found high concentrations of Fe, Cu and OC, in both “normal deceleration” and “full stop” braking patterns. Fe, Cu and Mn significantly correlated between each other suggesting their common brake wear origin. They also found that OC was present in one order of magnitude higher concentrations compared to EC (Riediker et al. 2008). Garg et al. (2000) found average trace element percent in total PM_10_ mass of 72 % with Fe, Cu, Ti, S and Zr being the most abundant. Over 96 % of the total carbon mass was attributed to organic substances in agreement with Riediker et al. (2008). Regarding the presence of Sb in airborne PM, existing studies seem to have reached contradictory conclusions. Some researchers reported the absence of Sb in airborne wear particles (Kukutschová et al. 2011; Wahlström et al. 2010a), which is supported by the intention of brake manufacture companies to substitute Sb in modern brake linings von Uexküll et al. (2005). On the other hand, Varrica et al. (2013) concluded that Sb is present in road dust and atmospheric PM samples in the form of Sb (III) and Sb (V). Sb_2_S_3_ was also detected in some of the ambient PM_10_ samples despite the fact that it is easily decomposed into more stable compounds during the brake abrasion process. Table 3 provides an overview of the concentrations of the most common elements found in brake wear dust and PM_10_. Sometimes, brake dust samples appear to have lower concentrations of trace elements compared to airborne PM, probably due to higher volatilization of organic constituents in airborne PM compared to dust samples (Sanders et al. 2003). Regarding specific organic compounds, Rogge et al. (1993) reported that only a small fraction of the organic content of brake dust could be extracted and analysed by conventional laboratory techniques. Among the organic compounds detected, polyalkylene glycol ethers (56.9 %) and n-alkanoic acids (34.3 %) were the most abundant species, while n-alkanes, PAHs and substituted PAHs were also detected in trace concentrations. In conclusion, although the presence of species like transition metals and carbonaceous compounds in wear particles is confirmed, there is still much information missing particularly regarding the organic constituents of wear particles.

**Table 3:** Trace element concentrations found in emitted brake wear dust

Metal	Brake dust (mg/kg)	Metal	Brake dust (mg/kg)
Al	330–20,000	Mg	(1700)–83,000
As	<2.0–(110)	Mn	620–5640
Ba	(5800)–140,000	Mo	5.0–740
Ca	500–8600	Na	80–(5100)
Cd	<0.06–11	Ni	80–730
Co	12–42.4	Pb	4.0–1290
Cr	135–12,000	Sb	4.0–19,000
Cu	70–210,000	Sn	230–2600
Fe	1300–637,000	Ti	100–110,000
K	190–39,000	Zn	120–27,300

Values in brackets refer to PM_10_ brake wear (Hildemann et al. 1991; Garg et al. 2000; Kennedy et al. 2002; Westerlund and Johansson 2002; Kennedy and Gadd 2003; Sanders et al. 2003; Von Uexküll et al. 2005; Schauer et al. 2006; Iijima et al. 2008)

## Brake wear PM emission factors

Emission factors (EFs) are used by researchers and regulating agencies as a tool to quantify the emission of a specified pollutant by an individual vehicle or a vehicle fleet mixture. They are functional relations that predict the quantity of a pollutant that is emitted per distance driven, energy consumed or amount of fuel used. EFs are typically derived for vehicle categories and depend on several parameters, the most important of which are the vehicle characteristics and emission control technologies, the type and quality of fuel used and the ambient and operating conditions (Franco et al. 2013). In order to determine EFs from brake wear, either direct measurement from the sources is employed, including real-world test conditions or laboratory experiments, or receptor modelling is applied. Although direct measurements provide EFs of a small number of vehicles, they have the advantage of being conducted under very well controlled conditions, even if in case of brake wear there is a difficulty in simulating real-life braking conditions. On the other hand, receptor modelling requires accurate knowledge of source composition and assumes that the sources specified are responsible for the species measured at the receptor (Barlow et al. 2007). A list of key tracers used by various researchers over the last decade for identifying brake wear is given in Table 4. Sternbeck et al. (2002) proposed the ratio of Cu/Sb (4.6 ± 2.3) as typical of brake wear particles but differences often appear in the literature due to variations in brake pad composition and site characteristics (Adachi and Tainosho 2004; Hjortenkrans et al. 2007; Pant and Harrison 2013).

**Table 4:** Overview of most common key tracers used for brake wear emission calculation

Reference	Tracer	Reference	Tracer
Sternbeck et al. 2002	Ba, Cu, Sb	Gietl et al. 2010	Ba, Cu, Fe, Sb
Adachi and Tainosho 2004	Ba, Ce, Cu, Fe, La, Sb, Ti, Y, Zr	Keuken et al. 2010	Cu
Schauer et al. 2006	Ba, Cu, Fe, Sb, Si, Zn	Amato et al. 2011	Cu, Cr, Fe, Sb, Sn, Zn
Hjortenkrans et al. 2007	Cd, Cu, Pb, Sb, Zn	Apeagyei et al. 2011	Cu, Ba, Fe, Mo, Ti, Zr
Harrison 2009	Ba, Cu	Duong and Lee 2011	Cu, Ni
Iijima et al. 2008	Sb	Song and Gao 2011	Sb, Cu, Fe, Pb
Tanner et al. 2008	Cu, Cd	Harrison et al. 2012	Sb, Cu, Fe, Pb
Bukowiecki et al. 2009a	Cu, Fe, Mo, Sb, Sn, Zn, Zr	Lawrence et al. 2013	Ba, Cu, Fe, Mn, Ni, Pb, Sb
Dongarra et al. 2009	Cu, Mo, Sb	Varrica et al. 2013	Sb

Regarding direct measurements, Garg et al. (2000) tested commercial pads used in light-duty vehicles and found PM_10_, PM_2.5_ and PM_0.1_ brake wear EFs of 2.9–7.5, 2.1–5.5 and 1.2–3.1 mg km^−1^ veh^−1^, respectively. The upper limit of the range for PM_10_ EFs was very close to the US EPA value of 7.9 mg km^−1^ veh^−1^ reported for light-duty petrol vehicles equipped with asbestos brakes (Boulter 2006). Sanders et al. (2003) also reported a relatively high average brake wear PM_10_ EF of 8.1 mg km^−1^ veh^−1^ for low-metallic, semimetallic and NAO brake pads (Table 5). Somewhat lower EFs were reported by Iijima et al. (2008) who found a PM_10_ value of 5.8 mg km^−1^ veh^−1^ and PM_2.5_ value of 3.9 mg km^−1^ veh^−1^ in a study conducted with NAO brake pads. Overall, brake wear EFs for light-duty vehicles (LDVs) deriving from direct measurements fall in the range of 3.0–8.0 mg km^–1^ veh^−1^ (PM_10_) and 2.1–5.5 mg km^−1^ veh^−1^ (PM_2.5_).

**Table 5:** Brake wear PM_10_ emission factors found in the literature for LDVs (mg km^−1^ veh^−1^)

Reference	Type of study	Emission factor
Garg et al. 2000	Brake dynamometer study	2.9–7.5
Sanders et al. 2003	Brake dynamometer study	8.1
Iijima et al. 2008	Brake dynamometer study	5.8
Rauterberg-Wulff 1999	Receptor modelling (highway–tunnel)	1.0
Abu-Allaban et al. 2003	Receptor modelling	0–80
Luhana et al. 2004	Receptor modelling	8.8
Bukowiecki et al. 2009a	Receptor modelling (urban street canyon)	8.0
Bukowiecki et al. 2009a	Receptor modelling (highway)	1.6
USEPA 1995	Emission inventory	7.9
Lükewille et al. 2001	Emission inventory	1.8–4.9
Boulter et al. 2006	Emission inventory (RAINS model)	3.8
Boulter et al. 2006	Emissions inventory (CEPMEIP model)	6.0
Boulter et al. 2006	Emissions inventory (MOBILE 6.2 model)	7.8
Barlow et al. 2007	Emission inventory	4.0–8.0
NAEI 2012	Emission inventory	7.0

Abu-Allaban et al. (2003) employed the CMB receptor model in order to determine brake wear EFs of LDVs and heavy-duty vehicles (HDVs) at roadside locations in the USA. They calculated PM_10_ EFs of 0–80 mg km^−1^ veh^−1^ for LDVs and 0–610 mg km^−1^ veh^−1^ for HDVs. The corresponding PM_2.5_ EFs were 0–5 and 0–15 mg km^−1^ veh^−1^. Higher brake wear EFs were observed at freeway exit sites, while brake emissions in highways and tunnels were negligible. Rauterberg-Wulff (1999) also reported very low PM_10_ brake wear EFs in tunnel studies (1.0 mg km^−1^ veh^−1^ for LDVs and 24.5 mg km^−1^ veh^−1^ for HGVs). PMF was used at the APART project, and the EFs obtained were better complied with those of direct measurements. More specifically, PM_10_ brake wear EFs in an urban street canyon were found to be 8.0 ± 4.0 mg km^−1^ veh^−1^ (LDVs) and 81 ± 39 mg km^−1^ veh^−1^ (HDVs). EFs calculated at a highway sampling location turned out to be significantly lower (1.6 ± 1.1 and 9.0 ± 7.0 mg km^−1^ veh^−1^ for LDVs and HDVs, respectively). PCA was employed during the PARTICULATES project, and a mean brake wear factor of 8.8 mg km^−1^ veh^−1^ was derived in case of passenger vehicles (Luhana et al. 2004). NAEI provided more generalized exhaust and non-exhaust EFs by combining hot exhaust, cold start and evaporative emissions for each main vehicle type in the UK fleet averaged overall all road types. They calculated a PM_10_ brake wear EF of 7.0 mg km^−1^ veh^−1^ (passenger cars), while for PM_2.5_, the corresponding value was 3.0 mg km^−1^ veh^−1^ (NAEI 2012). For LGVs, they found EFs of 11 mg km^−1^ veh^−1^ (PM_10_) and 4.0 mg km^−1^ veh^−1^ (PM_2.5_). Barlow et al. (2007) reported total brake wear debris emission factors of 10–20 mg km^−1^ veh^−1^ (LDVs) and 50–80 mg km^−1^ veh^−1^ (HDVs), with however 40 % of these becoming airborne (PM_10_). This corresponds to PM_10_ EFs of 4–8 mg km^−1^ veh^−1^ for LDVs and 20–32 mg km^−1^ veh^−1^ for HDVs. Finally, RAINS, CEPMEIP and MOBILE 6.2 models used brake wear PM_10_ EFs of approximately 3.8, 6.0 and 7.8 mg km^−1^ veh^−1^ for passenger cars, respectively. RAINS and CEPMEIP models also used PM_2.5_ EFs of approximately 2.2 and 6.0 mg km^−1^ veh^−1^, respectively (Berdowski et al. 2001; Boulter et al. 2006; CEPMEIP 2012). Concluding, brake wear EFs for LDVs deriving from modelling fall in the range of 1.0–8.8 mg km^−1^ veh^−1^ (PM_10_), depending on the site.

## Health relevance of brake wear particles

Several key factors (i.e. size distribution, agglomeration state, chemical composition, surface area, chemistry and charge) need to be taken into account when investigating wear particle toxicity (Oberdörster et al. 2005), among which particle size and chemical composition seem to be the most important. Many studies have demonstrated that particle size affects particle deposition in the respiratory tract (Kumar et al. 2013; Poepping and Ginda 2010; Pope et al. 2002; Samet et al. 2000). Coarse particles are mainly deposited in the upper respiratory tract (nose and throat), while ultrafine particles penetrate deep into the lungs (Poepping and Ginda 2010), thus posing hazards related to oxidative stress and inflammation (Balakrishna et al. 2009; Karlsson et al. 2005; Oberdörster et al. 2005). They can also enhance early atherosclerosis, partly due to their high content in redox chemicals and their ability to synergize with known proatherogenic mediators in the promotion of tissue oxidative stress (Araujo and Nel 2009). Other studies have shown that ultrafine particles may become blood-borne and translocate to other tissues such as the liver, kidneys and brain (Geiser and Kreyling 1999; Oberdörster et al. 2005), while experiments to animals have shown translocation of inhaled ultrafine particles to the brain (Tjalve and Henriksson 1999). A considerable fraction of brake wear particles lie into diameters smaller than 100 nm (Garg et al. 2000; Kukutschová et al. 2011; Mathissen et al. 2011), thus posing concerns regarding its potential adverse health effects. The World Health Organization reported that adverse health effects of inhalable PM are due to exposure over both short (hours, days) and long (months, years) terms and include respiratory and cardiovascular morbidity as well as mortality from cardiovascular and respiratory diseases and from lung cancer (WHO 2013). However, there are still no comprehensive studies directly linking brake wear PM with adverse effects on human health.

Chemical composition of particles can also be an important factor causing adverse effects on human health. Several PM_2.5_ constituents—attached to black carbon—have been seen as responsible for adverse impacts, with the most important being PAHs, metals and inorganic salts (WHO 2013). Transition metals such as Fe, Cu, Ni and Cr are important due to their potential to produce reactive oxygen species (ROS) and therefore oxidative stress in biological tissues. Oxidative stress occurs when antioxidant systems are overwhelmed by oxidative processes. Oxidative stress can also result from redox cycling by semiquinone radicals from organic compounds adsorbed on the particles and from ROS produced by activated macrophages (Karlsson et al. 2005). Metals such as Zn, Al and Pb can also influence the toxic effects of transition metals either by enhancing or by lessening their activity (Kelly and Fussell 2012). Brake wear emissions are characterized by high Fe and Cu concentrations and therefore should be considered even though available data don not allow quantification of specific health impacts on the population level. Apart from transition metals, there are other elements which have been linked to negative responses. Epidemiology studies revealed a high correlation between blood Pb and cardiovascular mortality and morbidity (Schober et al. 2006), while Cu and S have been associated with increased monthly mortality (Pope et al. 2007). Burnett et al. (2000) found an association of Fe, Ni and Zn in PM_2.5_ with short-term mortality, while they reported that the total effect of these components was greater than that of the mass alone. Ostro et al. (2007) linked fine particle Ca, Cu, Fe, Zn, Mn, Pb, Ti and V with daily mortality, while Hirshon et al. (2008) associated previous-day fine particle Zn concentrations with increased paediatric asthma cases. Toxicology studies in healthy volunteers have associated neutrophilic inflammation in the lungs with Fe and Se (Ghio et al. 2000; Huang et al. 2003). Fe particle agglomerates have been linked to inflammatory responses, decreased transferrin concentrations and increased concentrations of ferritin and lactoferrin in the blood (Ghio et al. 1998). Pulmonary injury and inflammation were connected to high concentrations of Fe, Cu, Ni, Pd and Zn (Ghio et al. 2004), while Schaumann et al. (2004) reported a higher inflammatory effect in the lungs of healthy volunteers following metal-rich particle (high levels of Zn, Cu, Ni and Ca) instillation compared with fractions with a lower metal content (Kelly and Fussell 2012). Sb_2_O_3_ has been classified as a possible human lung carcinogen by the International Agency for Research on Cancer (IARC 1989; Varrica et al. 2013) and as a “Class 3 Carcinogen” via dust inhalation according to 67/548/EC and amendments. Numerous studies have shown high concentrations of most of these metals in airborne brake wear particles (Furuta et al. 2005; Garg et al. 2000; Gasser et al. 2009; Gietl et al. 2010; Hjortenkrans et al. 2007; Kukutschová et al. 2011; Iijima et al. 2007; Pant and Harrison 2013; Sanders et al. 2003; Wahlström 2009); therefore, the possibility that such particles may induce adverse health impacts to humans should not be excluded.

Besides studies which investigate adverse health effects of PM, there are studies which have focused on potential adverse effects of brake wear particles. Gasser et al. (2009) exposed cells directly to freshly emitted wear particles in order to investigate their toxic effects on lung cells in vitro. Their results suggest that the metallic content of brake particles (specifically Fe, Cu and Mn) damage tight junctions both in “normal deceleration” and “full stop” braking pattern, probably through a mechanism involving oxidative stress. Brake wear particles derived from “full stop” braking also cause pro-inflammatory responses in lung cells, probably through a mechanism involving organic compounds and black carbon. This is enhanced by the study of Mazzarella et al. (2007) who also found a significant correlation between increased pro-inflammatory responses and high concentrations of carbon in the small particle size range. Riediker et al. (2004) linked adverse health responses to a particle source with a brake wear signature. They reported that fine particles originating from speed-changing traffic modulates the autonomic control of the heart rhythm, increases the frequency of premature supraventricular beats and elicits pro-inflammatory and pro-thrombotic responses in young, healthy, non-smoking men. They proposed that these health effects might be associated with the levels of Cu, which strongly increased under speed-changing traffic conditions and was associated with brake abrasion. Additionally, the researchers pointed out that long-term cardiovascular risk cannot be excluded, especially when considering the reported increase in myocardial infarction among professional drivers and the increase in mortality among people living near major roadways.

## Conclusions

The main points which can be drawn from the present literature study are summarized below:

- Exhaust and non-exhaust sources contribute almost equally to total traffic-related PM_10_ emissions. Brake wear has been recognized as one of the most important non-exhaust traffic-related source, with its relative contribution to non-exhaust traffic-related emissions ranging between 16 and 55 % and to total traffic-related PM_10_ emissions between 11 and 21 %.
- It is estimated that approximately 50 % of total brake wear is emitted as airborne PM_10_. The rest may deposit on the road or nearby or maybe attracted by the vehicle. The fate of bigger particles has not yet been well investigated.
- Several factors affect both physicochemical characteristics and generation rates of brake wear particles, making it very difficult to understand the generation mechanisms and study the properties of brake wear particles. Furthermore, there is a wide variety of sampling methodologies and measurement techniques which very often result in non-comparable results.
- Brake wear PM_10_ usually displays a unimodal mass size distribution with maxima between 2 and 6 μm. Particle number distributions of brake wear PM_10_ appear to be bimodal with both peaks lying within the fine mode. Most researchers report one peak of the distribution being among ultrafine particles (< 100 nm), while others find it at somewhat bigger sizes (approximately 300 nm)
- The most important chemical constituents of brake wear are Fe, Cu, Ba and Pb. Organic carbon is also present in significantly higher concentrations compared to elemental carbon. On the other hand, there is very limited information regarding specific organic constituents of brake wear PM_10_.
- Brake wear PM_10_ emission factors of 2.0–8.0 mg km^−1^ veh^−1^ for LDVs have been reported. Most studies find PM_10_ EFs of approximately 6.0–7.0 mg km^−1^ veh^−1^, which is very close to the standard for exhaust emissions of Euro 5/6 diesel vehicles. Brake wear PM_10_ EFs of HDVs are approximately one order of magnitude higher than of LDVs. Most commonly used key tracers of brake wear are Cu and Sb.
- Brake wear contains particles from all fractions involved in the respiratory function. Additionally, some constituents of airborne brake wear particles have been recognized as dangerous or potentially dangerous for the human health. However, there are no comprehensive studies linking brake wear particles with adverse effects on human health, while it is difficult to extrapolate animal and in vitro studies to humans.
